# Supplementary material for: Does a Vegetarian Diet Affect the Levels of Myokine and Adipokine in Prepubertal Children?
Source: J Clin Med. 2021 Sep 3;10(17):3995. doi: 10.3390/jcm10173995 (PMC8432473; doi:10.3390/jcm10173995)
Supplement: Supplementary file 1 [file jcm-10-03995-s001.zip › jcm-1299237-supplementary.pdf]

Supplementary Materials

Table S1. Multivariate generalized linear model of myostatin in the combined group of vegetarians and omnivores with main effect and 2-way interaction with the diet group.

Parameter	B	SE	95% CI		Test of Hypothesis	
			Lower	Upper	Chi-sq	*p*
(Constant)	0.4869	0.2284	0.0393	0.9345	4.545	0.033
Main effects						
Diet group – vegetarians	-0.3001	0.3160	-0.9194	0.3192	0.902	0.342
Omentin	0.0003	0.0001	0.0001	0.0005	13.149	0.000
Fat (%)	0.0383	0.0270	-0.0146	0.0911	2.017	0.156
Fat/lean	-2.0218	1.5759	-5.1106	1.0669	1.646	0.200
Total energy intake	0.0000	0.0002	-0.0003	0.0003	0.012	0.914
Dietary calcium	-0.0005	0.0002	-0.0010	0.0000	4.711	0.030
Dietary vitamin B12	-0.0749	0.0495	-0.1720	0.0221	2.292	0.130
Dietary vitamin D	-0.0016	0.0072	-0.0157	0.0124	0.051	0.821
Dietary vitamin E	0.0257	0.0108	0.0045	0.0469	5.653	0.017
2-way interactions diet group with:						
Omentin	0.0000	0.0001	-0.0003	0.0003	0.009	0.926
Fat (%)	0.0307	0.0302	-0.0284	0.0898	1.037	0.309
Fat/lean	-0.7448	1.7816	-4.2367	2.7472	0.175	0.676
Total energy intake	-0.0003	0.0002	-0.0007	0.0001	1.554	0.213
Dietary calcium	0.0003	0.0003	-0.0003	0.0009	1.127	0.288
Dietary vitamin B12	0.1289	0.0545	0.0222	0.2357	5.602	0.018
Dietary vitamin D	-0.0360	0.0146	-0.0647	-0.0074	6.070	0.014
Dietary vitamin E	-0.0100	0.0171	-0.0435	0.0235	0.341	0.559
(Scale)	0.0710	0.0105	0.0533	0.0951		

Omnivores as reference category; B – unstandardized regression coefficient; SE - standard error; p - significance level; chi-sq test with 1 degree of freedom.

Table S2. Multivariate generalized linear model of irisin in the combined group of vegetarians and omnivores with main effect and 2-way interaction with the diet group.

Parameter	B	SE	95% CI		Test of Hypothesis	
			Lower	Upper	Chi-sq	*p*
(Constant)	0.9569	0.2212	0.5235	1.3904	18.720	0.000
Main effects						
Diet group – vegetarians	-0.3792	0.3177	-1.0018	0.2435	1.425	0.233
Omentin	-0.0001	0.0001	-0.0003	0.0001	1.379	0.240
BMC	-0.0010	0.0003	-0.0015	-0.0004	11.354	0.001

Total energy intake	0.0004	0.0002	0.0000	0.0007	4.727	0.030
Dietary manganese	-0.0337	0.0370	-0.1062	0.0388	0.830	0.362
2-way interactions diet group with:						
Omentin	0.0005	0.0002	0.0002	0.0008	9.580	0.002
BMC	0.0010	0.0004	0.0003	0.0017	7.608	0.006
Total energy intake	-0.0003	0.0002	-0.0007	0.0001	1.778	0.182
Dietary manganese	-0.0718	0.0565	-0.1825	0.0389	1.617	0.204
(Scale)	0.0970	0.0143	0.0724	0.1291		

Omnivores as reference category; B – unstandardized regression coefficient; SE - standard error; p - significance level; chi-sq test with 1 degree of freedom.
